# MORC3 represses the HCMV major immediate early promoter in myeloid cells in the absence of PML nuclear bodies

**DOI:** 10.1002/jmv.29227

**Published:** 2023-11-27

**Authors:** Anna Champion, Alexandra Rowland, Levia Yee, Dick van den Boomen, Matthew Reeves, Paul Lehner, John Sinclair, Emma Poole

**Affiliations:** ^1^ Department of Medicine University of Cambridge Cambridge UK; ^2^ Divison of Virology, Department of Pathology University of Cambridge Cambridge UK; ^3^ Department of Pathology University of Cambridge Cambridge UK

**Keywords:** human cytomegalovirus, latency, MORC3

## Abstract

Human cytomegalovirus (HCMV) can undergo either a latent or a lytic infection in cells of the myeloid lineage. Whilst the molecular mechanisms which determine the outcome of infection are far from clear, it is well established that a key factor is the differential regulation of the major immediate early promoter (MIEP) responsible for driving lytic immediate early gene expression. Using a myelomonocytic cell line stably transduced with a GFP reporter under the control of the MIEP, which recapitulates MIEP regulation in the context of virus infection, we have used an unbiased CRISPR‐Cas9 sub‐genomic, epigenetic library screen to identify novel cellular factors involved in MIEP repression during establishment and maintenance of latency in myeloid cells. One such cellular factor identified was MORC3. Consistent with MORC3 being a robust repressor of the MIEP, we show that THP1 cells devoid of MORC3 fail to establish latency. We also show that MORC3 is induced during latent infection, recruited to the MIEP and forms MORC3 nuclear bodies (MORC3‐NBs) which, interestingly, co‐localize with viral genomes. Finally, we show that the latency‐associated functions of MORC3 are regulated by the deSUMOylase activity of the viral latency‐associated LUNA protein likely to prevent untimely HCMV reactivation.

## 1 INTRODUCTION

Human cytomegalovirus (HCMV) is a ubiquitous beta‐herpesvirus that infects up to 99% of individuals in certain populations, depending on demographics. The virus is generally asymptomatic in immune competent individuals but causes a major disease burden in the immune compromised/suppressed, such as transplant patients, or in immune naïve neonates.^1^, ^2^, ^3^ This is partly explained by the fact, after primary infection, virus establishes a lifelong latent infection in the host during which time the production of infectious virions is undetectable. However, periodically the virus is able to reactivate from latency resulting in infectious virion production though such reactivation events are usually subclinical due to robust immune control.^4^, ^5^, ^6^ One well‐recognized site of HCMV latency is undifferentiated cells of the myeloid lineage, such as CD34+ and CD14+ cells, and as these cells differentiate into terminally differentiated cell types, such as dendritic cells and macrophages, the virus reactivates.^4^, ^7^ Whilst the exact mechanisms which govern latency and reactivation are not fully understood, it is known that the major immediate early (IE) promoter (MIEP) plays a critical role in this process; it drives expression of key lytic genes, encoding the IE proteins, during lytic infection and, conversely, is strongly repressed during latency.^8^, ^9^, ^10^, ^11^

This latency‐associated suppression of the MIEP is known to result from the recruitment of repressive factors, such as YY1,^12^ and subsequent recruitment of histone deacetylases (HDACs) and histone methylases^13^, ^14^ resulting in marks of repressed chromatin on the MIEP during latent infection. However, the full spectrum of transcription factors involved in deposition of these repressive marks on the MIEP during latency is unclear. To further understand the mechanisms which regulate HCMV latency and reactivation, we carried out a sub‐genomic, epigenetic library CRISPR/Cas9 screen to identify cellular transcription factors which repress the MIEP in stably transfected THP1 cells carrying the full length viral MIEP controlling expression of GFP. Using this approach a number of factors which were involved in MIEP repression were identified. Three of these were chosen for validation and one, microrchidia family CW‐type zinc finger 3 (MORC3), was pursued in the context of full virus infection and mechanism of action.

There are five human MORC family members characterized by a GHKL (Gyrase B, Hsp90, histidine kinase and MutL)‐ATPase at the N terminus; a central CW‐type zinc finger (CW) domain and, at the C terminus, a coiled‐coil domain containing five SUMOylation sites. MORC proteins can dimerize via the ATPase (ATP‐dependent) domain as well as the coiled‐coil domain (constitutively).^15^, ^16^ Dysregulation of MORC3 (and other MORC proteins) has been associated with cancer and disease.^17^ MORC3 was originally shown to play a role in modification of chromatin via direct binding to activatory H3K4me3 as well as repressive H3K9me3^18^ and recognizes these epigenetic modifications by its CW domain. Functionally, MORC3 has been demonstrated to play a repressive role in several settings and the extent of SUMO modification of MORC3 appears to impact on the mechanism by which repression occurs. For example, SUMOylated MORC3 can mediate transcriptional repression by complexing with PML to form Daxx containing NBs.^19^, ^20^ In contrast, deSUMOylated MORC3 forms NBs independently of PML and forms a repressive molecular DNA clamp.^20^ In herpes simplex virus 1 (HSV‐1) or HCMV lytic infection, MORC3 is known to play a repressive role in a Daxx‐dependent manner and is targeted for degradation to enhance viral lytic gene expression.^21^, ^22^ Furthermore, it has also been demonstrated that MORC3 silences endogenous retroviruses again in a Daxx dependent manner. Thus, MORC3 regulates both integrated and episomal viral DNA genomes.^23^ We now show that, in contrast to lytic infection,^24^ MORC3 is upregulated during latent infection. Importantly, MORC3 is desumoylated in latently infected cells by a virally encoded deSUMOylase, LUNA. This results in an increased accumulation of MORC3‐containing NBs promoting co‐localization of MORC3 with HCMV genomes and, ultimately, leads to a Daxx‐independent repression of the MIEP.

## 2 METHODS

### 2.1 Viruses and cells

THP1 cells were maintained in RPMI supplemented with 10% FCS and 1% penicillin/streptomycin (Gibco). Human foetal foreskin fibroblasts (HFFFs) and Retinal Pigment Epithelial‐1 (RPE‐1) cells and 293‐T cells were cultured in DMEM‐10 supplemented with 10% FCS and 1% penicillin/streptomycin (Gibco). All cells were maintained in 5% CO_2_ and at 37°C.

Myeloid cell differentiation and virus reactivation assays, including fibroblast co‐culture assays were carried out as previously described.^25^

Primary monocytes were isolated from venous blood as described previously. HCMV TB40E‐derived viruses were used which have been described previously: TB40E‐IE2YFP, which expresses YFP fused to the immediate early gene IE2 (IE86) and TB40E‐GATA2mCherry which expresses mCherry from the GATA2 promoter.^13^, ^25^, ^26^, ^27^ Edu labeled virus was generated by infecting RPE‐1 cells with TB40E‐SV40GFP at MOI‐1 in the presence of 2 µM EdU and harvested at days 5–9. Optimal virus was generated at 6 days postinfection. Harvests were pooled and concentrated twice with washing in between to remove any unassociated EdU (which otherwise integrates into host cell genomes). Concentrated virus was resuspended in X‐Vivo15 and titrated on RPE‐1 cells to determine myeloid tropism. Lentivirus to generate small hairpin RNA (shRNA) MORC3 depleted cells was obtained from Santa Cruz.

MIEP‐GFP expressing THP1 cells were generated using lentiviral transduction. First cells were lentivirally transduced with Cas9 and selected with blastocydin. Next, Lentivirus plasmid (pLV) containing the MIEP fused to GFP was generated with 2057 nucleotides of the MIEP (nucleotides 174 041–176 097 Human herpesvirus 5 strain Merlin isolate RCMV2035, Genbank ID: KP973642.1) encompassing all enhancer modulator and repressor regions was generated.^28^ This plasmid was co‐transfected alongside lentiviral plasmids PsPax and PMD2.G into 293T cells using mirus293 transfection reagent (Mirus™) in accordance with the manufacturer's guidelines. After 48 h supernatants were harvested, filtered (0.4 nm filtration) and then used to transduce THP1 cells. Transduced cells were selected with puromycin.

### 2.2 Library lentiviral transduction

Stable protein expression was achieved using lentiviral transduction. 293T cells were co‐transfected in a 1:1 ratio with a lentiviral expression vector (pHRSIN/pHRSiren/pKLV) and the packaging vectors pMD.G and pCMVR8.91 using TransIT‐293 (Mirus). Supernatant was harvested at 48 h posttransfection and transferred to target cells. Cells were incubated with virus overnight. Transduced cells were selected for stable transgene expression with appropriate antibiotics from 48 h posttransduction.

### 2.3 Generation of epigenetic library

We generated an epigenetic library containing genes necessary for the regulation, initiation, maintenance or removal of epigenetic modifications to the genome. The candidate gene list for the library was assembled by integrating multiple source lists of known epigenetic regulators.^29^, ^30^, ^31^ Candidates appearing on multiple lists were immediately accepted. Other candidate genes were manually reviewed for inclusion, resulting in a total number of 1428 genes in the library. Single guide RNA (sgRNA) sequences were selected by filtering high‐performance libraries for unique sgRNA sequences.^32^, ^33^, ^34^ Where possible, five individual sgRNA were selected per gene, with a total of 7108 sgRNAs in the library and additional control pool of 340 non‐targeting sgRNAs.^35^

### 2.4 CRISPR/Cas9 epigenetic library screen

The epigenetic library was cloned from a single oligo DNA chip in two rounds of PCR. Round one amplified the sgRNAs with adaptor sequence. Round two removed the adaptor and added a complementary overhang to the digested vector. PCR product was run on a 0.7% agarose gel and extracted using QIAEX2 kit (20021, Qiagen). Plasmid pKLV‐U6gRNA (BbsI)‐PGKpuro2ABFP (#50946, a gift from Kosuke Yusa, Addgene plasmid) was modified from with the addition of a small BsmBI stuffer sequence to allow restriction digest with BsmBI (NEB R0739S, manufacturers protocol) into the U6 promoter region and tracrRNA. The digested vector was gel purified as above. The resulting DNA oligo was inserted into the prepared plasmid backbone using HIFI assembly (NEB E2621, manufacturer protocol). The completed reaction was purified using AmpureXP (Beckman) at a ratio of 0.75:1. Electrocompetent *E. Coli* (DH10B Thermo EC0113) were electroporated with the assembled plasmid and allowed to grow‐out for 1 h at 32 degrees in LB‐Amp media. Media was then plated on LB‐Amp plates with controlled serial dilutions to allow estimate of library coverage. Following overnight incubation, colonies were counted and collected. Purified plasmid DNA was obtained by DNA extraction using midi‐prep (740420, Macherey Nagel).

PCR primer sequences for CRISPR library screen

**Table:** 

PCR1 FWD	GAATGAACATGACGCTGGGA
PCR1 REV	CACCTTCAATGATGACCCCA
PCR2 FWD	ccgtaacttgaaagtatttcgatttcttgGCTTTATATATCTTGTGGAAAGGACGAAACACC
PCR2 REV	caagttgataacggactagccttatttaaaCTTGCTATGCTGTTTCCAGCATAGCTCTTAAAC

### 2.5 Genome‐wide and targeted sub‐genomic CRISPR screening

THP‐1 MIEP GFP cells were stably transduced with Cas9. CRISPR sgRNA library lentivirus was produced as indicated above and titrated on THP‐1 MIEP GFP CAS9 cells. For targeted CRISPR screening 20 million THP‐1 MIEP GFP CAS9 were transduced at 35% infectivity with the epigenetic sgRNA library, sorted at day 8. 1.5% (300 000 cells) of the brightest BFP+/GFP^high^ phenotype and genomic DNA was extracted from sorted cells alongside an age‐matched library sample.

Integrated sgRNA was amplified via two rounds of PCR using primers as indicated in Supporting Information materials, starting with 50 μg of gDNA for the library and sorted sample. PCR products were purified using AMPure XP beads (Agencourt), quantified on a DNA‐1000 chip (Agilent) and sequenced on a Miniseq sequencer (Illumina) running MiniSeq Control Software v1.1.8 (Illumina). Reads were aligned to library sequences using Bowtie,^1^ allowing read alignment to a maximum of two sgRNA and one mismatch. sgRNA abundance in the sorted sample was compared against a screen‐internal library sample and sgRNA enrichment was computed using the MAGeCK algorithm under default settings.^2^ Hits with a MAGeCK score <10^−5^. Full datasets, including read counts, MAGeCK analysis, and selected hits, are available in Supporting Information Data.

### 2.6 Visualization of EdU labeled virus

Click Chemistry was used to visualize EdU labeled genomes. The Click‐iT EdU 594 kit (Invitrogen) was used following the manufacturer guidelines.

### 2.7 Proteomic and RNAseq screens

The original unbiased proteomic and RNAseq analyzes have been published.^13^, ^27^

### 2.8 FACS

THP1 cell lines were stained for MHC‐1 (W6/32 antibody for MHC‐I from Lehner Laboratory hybridoma) and prepared for FACS analysis as described previously.^26^

### 2.9 Chromatin immunoprecipitation (ChIPs)

ChIPs were carried out using the Imprint ChIP kit (Sigma) with antibodies anti‐histone H3 (Upstate) and anti‐MORC3 (Santa Cruz) using the manufacturer protocol.

### 2.10 Immunofluorescence

PML was detected with anti‐PML‐488, 546, and 647 and anti‐MORC3‐488, 546, and 647 (Santa Cruz) were used in different combinations depending on the experiment. Nuclei were visualized with Hoechst 33342. Cells stained for EdU as well as proteins were stained using the Click‐iT protocol for multi‐staining where the cells were stained for EdU first and then co‐stained with antibodies and Hoechst as described in the kit protocol. For staining without EdU, cells were fixed in 1% paraformaldehyde (20 min, Sigma) and permeabilised in 0.01% Triton X‐100/PBS (15 min, Sigma) before blocking in 3% bovine serum albumin (BSA)/phosphate buffered saline (PBS) (30 min, Sigma). Antibodies were used at 1 in 250 in 3% BSA/PBS. Cells were washed 3× PBS in between each step and visualized either directly or after mounting on slides in Citifluor.

### 2.11 Immunoprecipitation

CD14+ cells (5 × 10^7^ per sample) were infected with the indicated viruses for 4 days and then harvested for immunoprecipitated as described previously with the addition of complete protease inhibitor (Roche). Samples were then analyzed by western blot.

## 3 RESULTS

### 3.1 Identification of MORC3 as a repressive epigenetic factor of the MIEP using an unbiased screen

**Figure 1:**
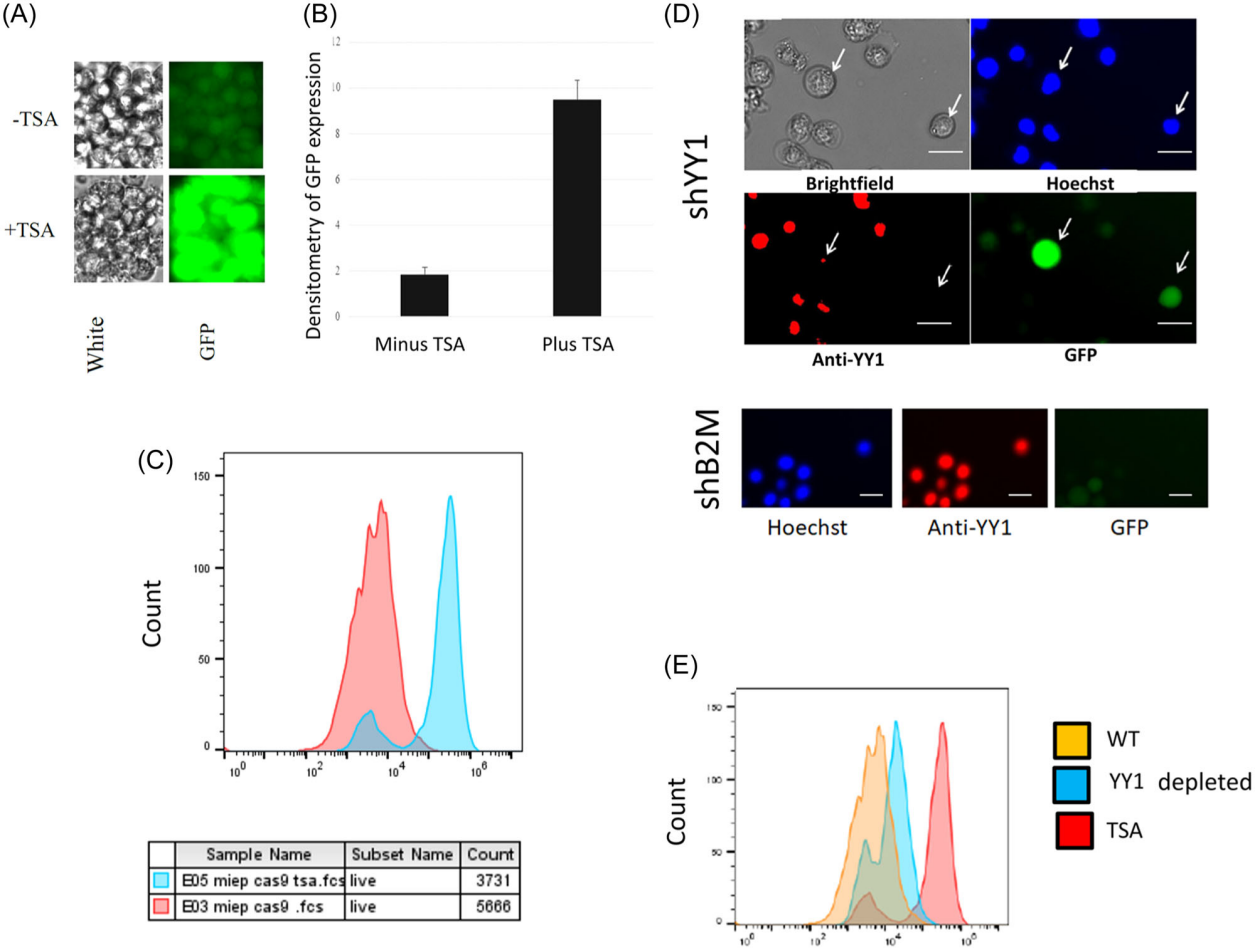
As with HCMV latently infected cells, the MIEP in MIEP‐GFP THP1 cells is activated by the addition of HDACi or depletion of YY1. THP1 cells were stably transduced with MIEP‐GFP (MIEP‐GFP THP1) and selected with puromycin before either adding TSA and evaluating levels of GFP expression by fluorescence (A), densitmetry of fluorescence (B) or FACS (C). Alternatively, MIEP‐GFP THP1 cells were transduced with shYY1 lentivirus and then assessed for the presence of YY1 and concomitant GFP expression by fluorescence where the white line represents 20 µM (D) and FACS (E). HCMV, human cytomegalovirus; MIEP, major immediate early promoter.

Although it is known that the MIEP is associated with marks of repressive transcription during HCMV latency, the full epigenetic control of the MIEP is not understood. To gain further insight to how the MIEP is controlled in cells which support HCMV latency, we generated a myelomonocytic cell line (THP1) stably expressing the MIEP driving the expression of GFP. These undifferentiated myeloid THP1 cells support a “latent” HCMV infection with reduced IE gene expression in the absence of production of virions and can be differentiated into terminally differentiated myeloid cells where HCMV reactivates. Therefore, these cells recapitulate the HCMV latency and reactivation observed in primary myeloid cells. Whilst this reductionist approach to interrogation of the MIEP (ie, the MIEP in the absence of the rest of the virus) has the potential to be very informative, we first ensured that basal expression of the integrated MIEP‐reporter in undifferentiated THP1 was responsive to the same activatory and repressive stimuli as the MIEP in the context of the latent viral genome. Figure 1 shows that the integrated MIEP‐reporter can be derepressed with the HDAC inhibitor TSA (Figure 1A–C), exactly as seen in latently infected myeloid cells.^36^ Conversely, depletion of YY1, a known cellular transcriptional repressor of the MIEP, stimulates the integrated MIEP‐reporter (Figure 1D,E). Together, these data suggested that THP1 cells carrying an MIEP‐reporter were suitable as a model for the identification of factors which activate or repress the MIEP in undifferentiated myeloid cells.

**Figure 2:**
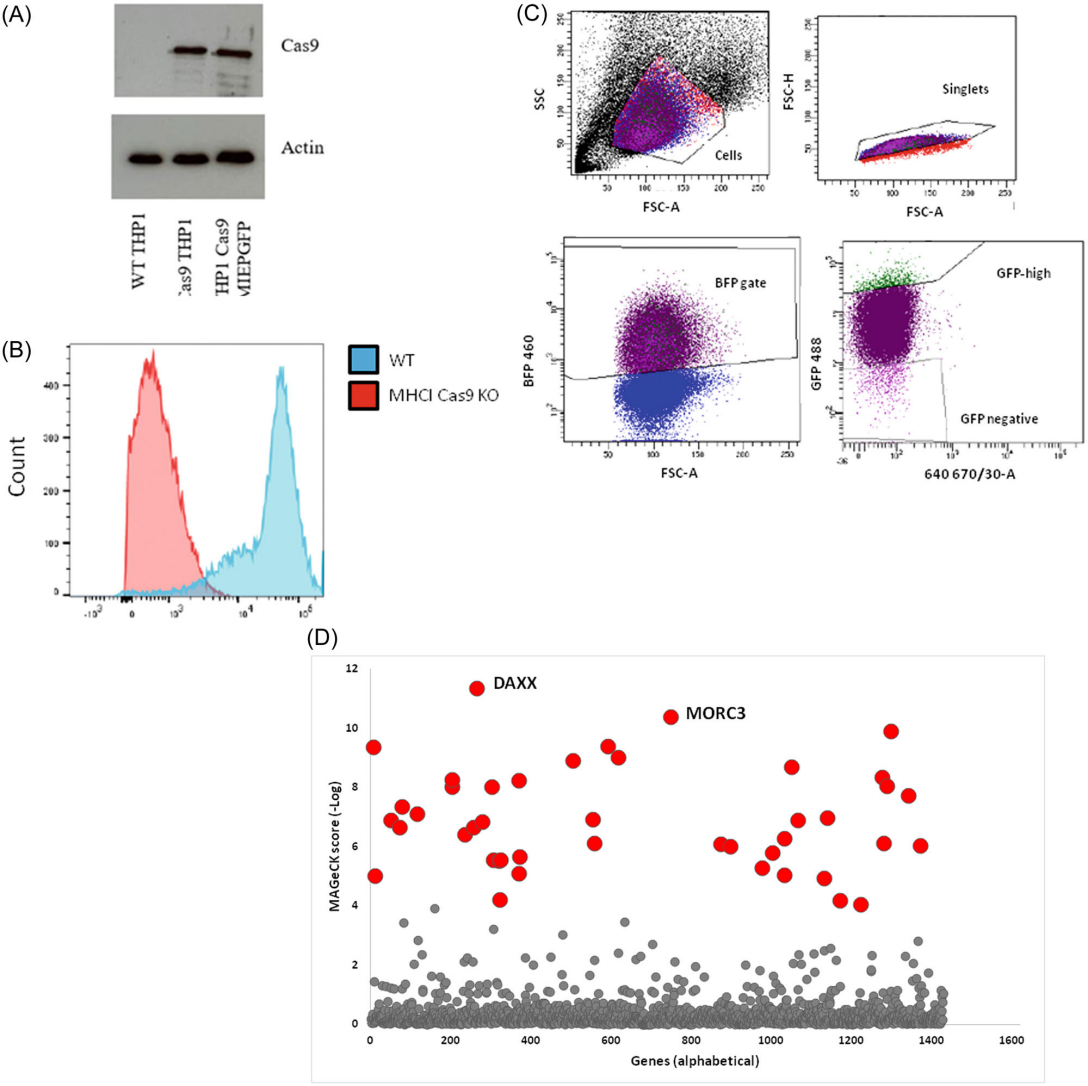
MIEP‐GFP reporter THP1 cells transduced with Cas9 express functional Cas9 and enable an unbiased CRISPR/Cas9 screen of MIEP regulators. MIEP‐GFP reporter THP1 cells were transduced with Cas9 and analyzed for Cas9 expression by western blot (A). Functional Cas9 expression was assessed by subsequent lentiviral delivery of guide RNAs to MHC class 1. Cell surface MHC class 1 expression was then assessed by FACS (B). A gRNA library targeting epigenetic transcription factors in lentiviruses carrying a blue fluorescent protein (BFP) marker was also introduced and BFP positive cells with increased GFP fluorescence were selected (C). DNA was isolated and illumina sequencing carried out to identify CRISPR/Cas9 targets. The graph represents proteins which, when removed by CRISPR/Cas9, resulted in significantly higher GFP fluorescence in MIEP‐GFP reporter cells (D). gRNA, guide RNA; MIEP, major immediate early promoter.

To identify potential epigenetic factors which may play a repressive role on the MIEP, we undertook a CRISPR/Cas9 screen using an epigenetic library. To do this, we stably transduced Cas9 into the MIEP‐reporter cells by lentiviral transduction. Figure 2A,B show that Cas9 is expressed in the MIEP‐reporter THP1 cells and that it is functional. Only cells transduced with Cas9 showed detectable levels of Cas9 by western blot (Figure 2A) and when a lentivirus containing guide RNAs to b2M were introduced to the Cas9 expressing cells, cell surface MHC class 1 expression was decreased (Figure 2B) confirming a functional Cas9. Next, these cells were used for a screen of epigenetic regulators which transactivate the MIEP. The resulting lentiviruses carried a blue fluorescence protein (BFP) expression cassette for selection. Figure 2C shows the sorting gates for cells co‐expressing BFP and high levels of GFP (which would be indicative of de‐repression of the MIEP promoter). This enriched cell population was sequenced by Illumina Sequencing to identify the differentially regulated guide RNA. The top hit from the screen was Daxx, a known transcriptional repressor of the MIEP thus acting as a validatory “positive control” for the screen approach. Our next top hit was MORC3 which was selected for further study.

### 3.2 Validation of MORC3 as a repressive factor for the MIEP

**Figure 3:**
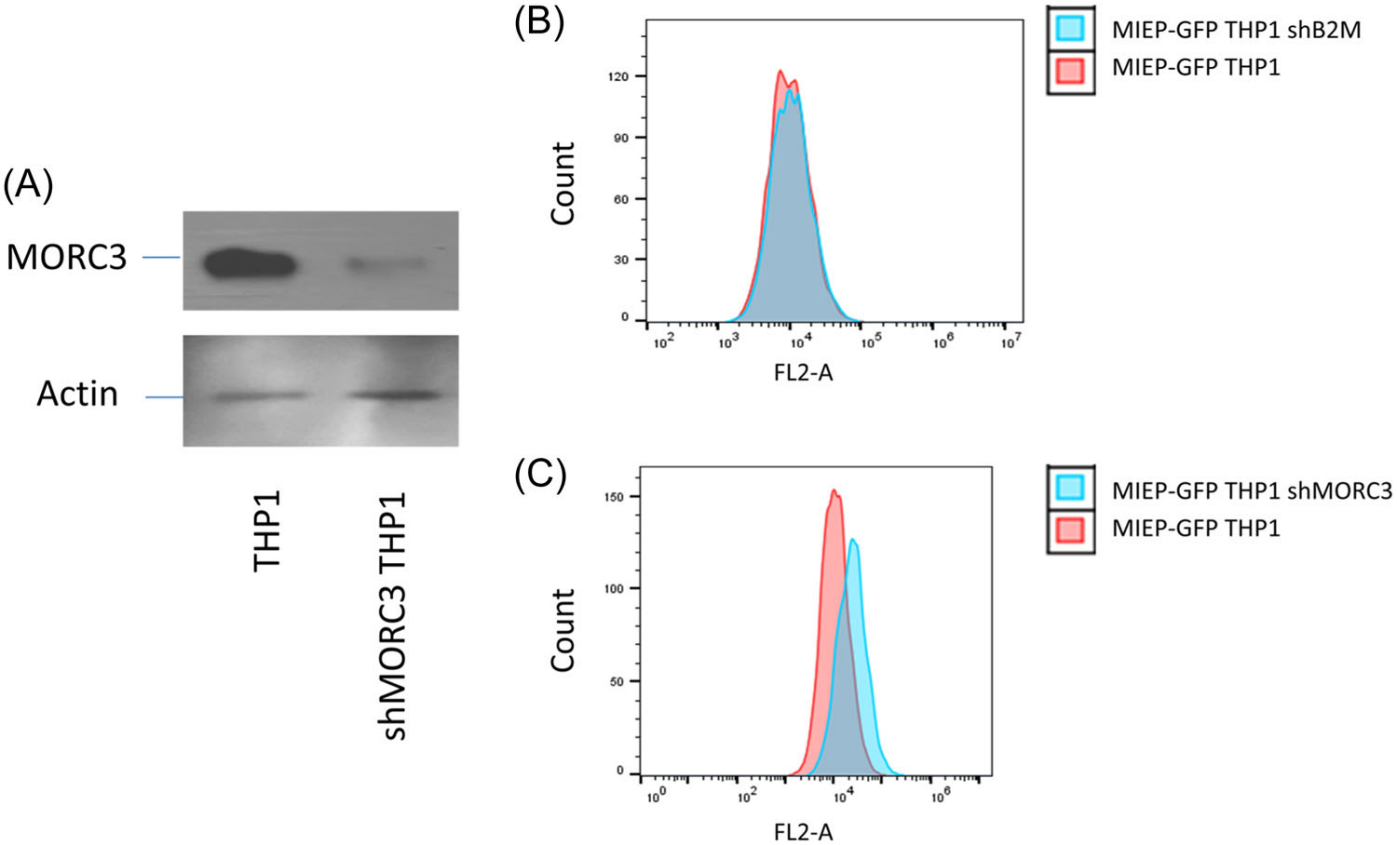
Removal of MORC3 leads to an increase in GFP expression in THP1 MIEP‐GFP cells. THP1 cells were transduced with shMORC3 lentivirus and analyzed for MORC3 and actin levels by western blot (A). Alternatively, THP1 cells stably expressing MIEP‐GFP were either left untransduced or transduced with shB2M (B) or shMORC3 (C) for 7 days before analyzing for GFP expression by FACS. MIEP, major immediate early promoter.

MORC3 is a cellular epigenetic factor whose expression is reduced during lytic infection for optimal IE gene expression.^21^ Therefore, we asked whether MORC3 was required for the establishment and maintenance of latency. First, the ability of MORC3 to act as a repressive factor for the MIEP was analyzed. To do this, we knocked down MORC3 expression in MIEP‐GFP reporter cells by lentivirus transduction and confirmed MORC3 knockdown in comparison to cells after delivery of an irrelevant shRNA targeting B2microglobulin (Figure 3A). We next compared the levels of GFP expression in untreated MIEP‐GFP reporter cells, cells transduced with shRNAs to B2microglobulin (shB2M) and cells transduced with shMORC3. Figure 3B,C show that removal of MORC3 led to an increase in MIEP derived GFP expression, validating the screen and confirming that MORC3 acts as a repressor of the HCMV MIEP.

### 3.3 MORC3 is upregulated during viral latency but downregulated during monocyte differentiation

**Figure 4:**
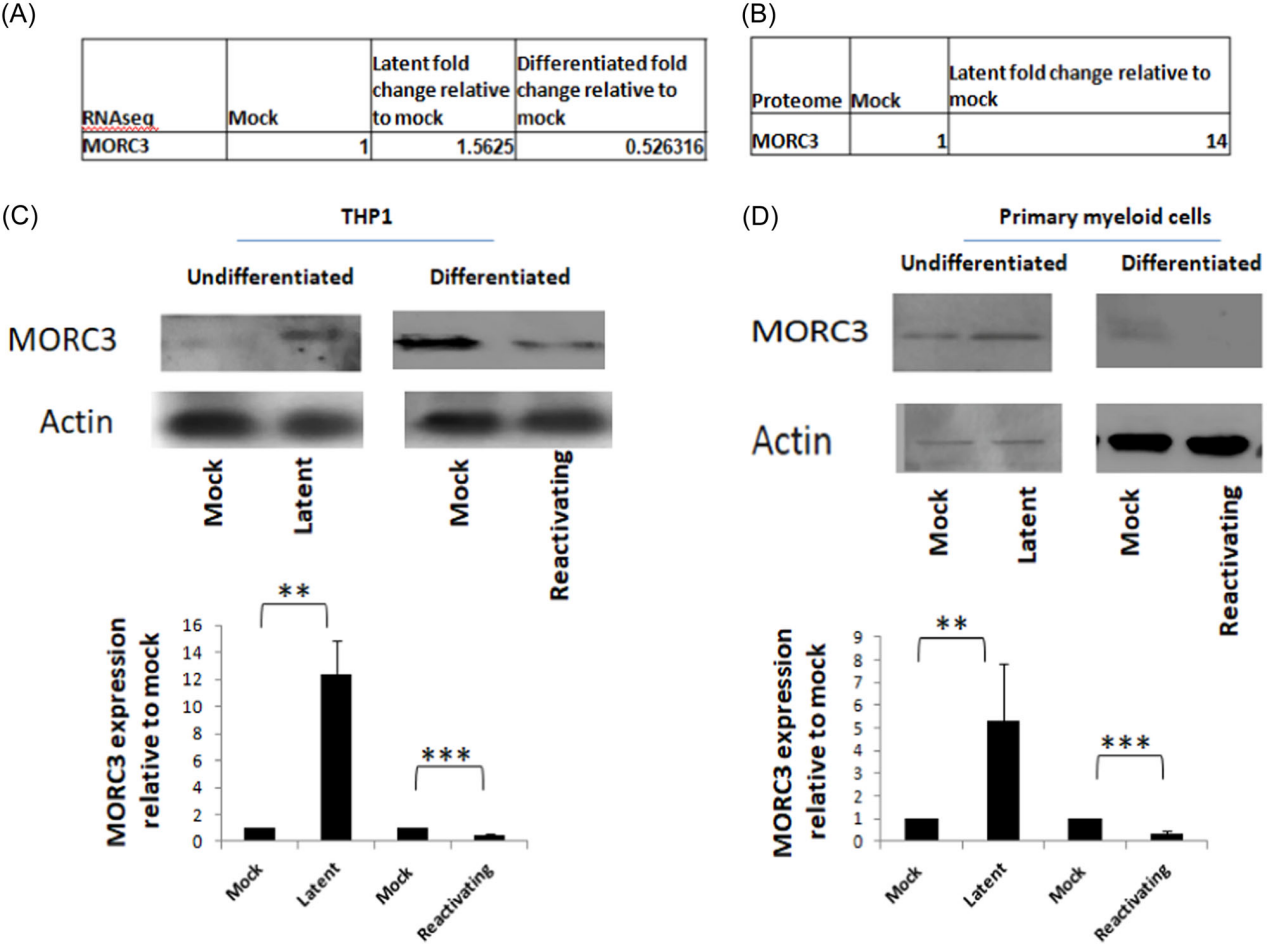
Latently infected cells upregulate MORC3 whereas reactivating cells decrease MORC3. Reanalysis of RNAseq and proteome data (A and B). Primary CD14+ monocytes were either uninfected or infected with TB40E‐GATA2mCherry and then differentiated into dendritic cells or left undifferentiated and harvested for RNAseq. Values shown are levels of MORC3 RNA relative to mock infected cells (A). Alternatively CD14+ primary monocytes were infected with TB40E‐SV40GFP for 2 days before sorting and then cultured for a further 4 days before being harvested for full proteomic analysis (B). THP1 cells were latently infected with TB40E SV40GFP virus for 2 days before sorting for GFP positivity. Sorted cells were then left for a further 2 days. After this, sorted cells were left untreated, differentiated for a further 2 days with PMA and then harvested for western blot analysis of actin and MORC3 proteins (C). The same experiment as (C) was carried out but using primary CD14+ monocytes and the cells were differentiated with GM‐CSF/IL‐4 and LPS instead of PMA (D). Data for C and D shows an example western blot and densitometry of triplicate samples for statistical analysis where graphs represent standard deviation about the mean with the Student *t*‐test significance (**p < 0.005, ***p < 0.0005). HCMV, human cytomegalovirus; MIEP, major immediate early promoter.

Although MORC3 was identified and confirmed to be a repressive factor for regulation of the MIEP‐GFP when integrated in our stably transduced THP1 cells, we wanted to confirm this in the context of latent virus infection. Initially to investigate this, we reanalyzed our previously published single cell RNAseq data detailing changes in cellular gene expression during latent carriage and reactivation of primary myeloid cells.^13^ This showed that there was a modest but clear increase (1.5‐fold) in MORC3 RNA during latency and a relative decrease (twofold) of MORC3 RNA upon cellular differentiation and reactivation (Figure 4A). Whilst these changes are small, the decreases in MORC3 RNA in infected, differentiated cells is consistent with previous reports that MORC3 is downregulated during HCMV infection in differentiated cells.^13^, ^21^ Similarly, a reanalysis of a latency‐associated proteome analysis we had previously carried out^27^ (Figure 4B) also showed that MORC3 protein was upregulated by 14‐fold in latently infected primary monocytes, suggesting that MORC3 could be an important target for HCMV latency.

We next validated these data by western blot and densitometry (to enable statistical analysis). Figure 4C shows that sorted latently infected THP1 cells have increased levels of MORC3 protein relative to uninfected cells and conversely, differentiated THP1 cells have reduced levels of MORC3 protein when infected with HCMV. We also carried out this analysis with primary monocytes and also observed increased levels of MORC3 in latently infected monocytes but reduced levels of MORC3 in infected differentiated monocyte (Figure 4D, data shows an example of a western blot and statistical analysis by densitometry). Together these data show that MORC3 is upregulated during HCMV latency, in contrast to being downregulated during reactivation/differentiation.

### 3.4 Identification of MORC3 as a repressive epigenetic factor for HCMV latency

Having established that MORC3 has a repressive effect on the MIEP driving GFP in stably transduced THP1 cells and that MORC3 is upregulated during latency in both THP1 cells and primary monocytes, we next investigated whether MORC3 also plays a repressive role on the MIEP in the context of a latent infection.

**Figure 5:**
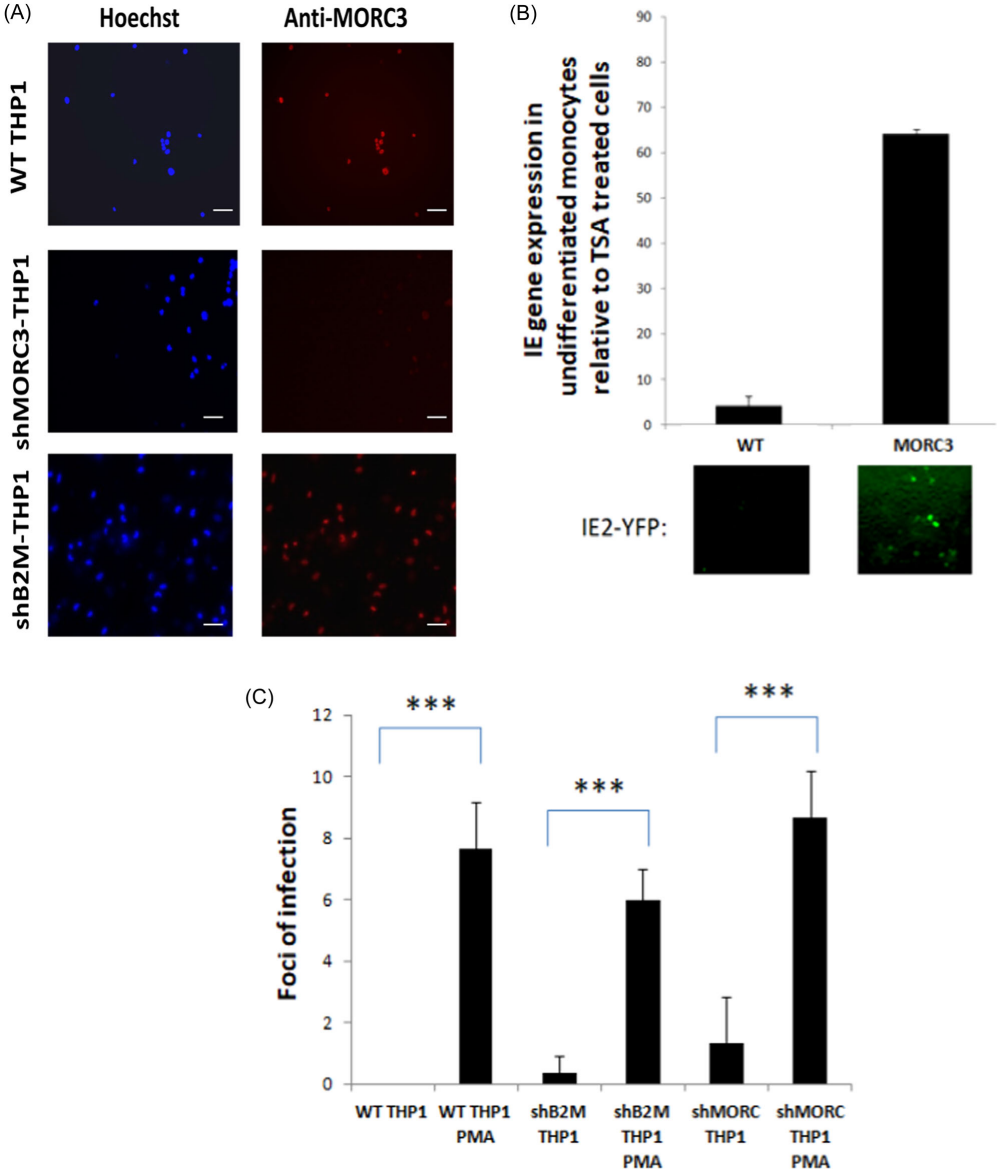
The presence of MORC3 is required for optimal repression of IE during latency. Untreated THP1 cells or THP1 cells transduced with shMORC3 lentivirus were stained for MORC3 (red) or hoechst 33342 (blue) where the white line represents 50 µM (A). These cells were then infected with TB40E‐IE2YFP virus for 4 days before enumerating for IE positive nuclei by fluorescence microscopy. The same cells were treated with TSA to reactivate IE gene expression in any latently infected cells in the population. The graph represents numbers of IE positive cells in three fields of view of uninduced cells relative to TSA treated cells with images from cells shown below the graph (B). Alternatively these cells were treated as for (B) except that after 24 h of differentiation the fully differentiated cells were cultured for 5 days instead of treating with TSA. Cells were then freeze thawed to release any cell associated virus before transferring to HFFFs. Foci of infection resulting from these supernatants were enumerated. Triplicate samples with standard deviation about the mean (B) and the Student *t*‐test significance (***p ≤ 0.0001) are shown (C). IE, immediate early.

To do this we knocked down MORC3 in THP1 cells using shMORC lentiviral transduction and confirmed reduced MORC3 levels by immune fluorescence (Figure 5A). We then analyzed these cells for their ability to suppress IE gene expression in the context of a latent infection by infecting them with TB40E‐IE2YFP virus—as this virus expresses IE2 protein fused to YFP, lytically infected cells are easily identifiable. Figure 5B shows that in the absence of MORC3, THP1 cells are unable to repress the MIEP effectively, resulting in high numbers of cells expressing IE2YFP after infection, relative to TSA treated cells (which is a known to cause derepression of IE expression in latently infected cells and indicates the number of reactivatable cells in the population which allows relative reactivation in the absence of MORC3 to be calculated relative to a known strong differentiation stimulus). These data argue that removal of MORC3 before infection prevents the establishment of latency.

Next, to test whether the shMORC cells that fail to establish latency support a full productive infection, WT THP1 cells or shMORC3 THP1 cells infected for 4 days were treated with PMA for 24 h before transferring cell supernatant to fibroblasts to determine virus production. Figure 5C shows that compared to PMA treated cells, infection of MORC3 knockdown cells which fail to establish latency does not result in full virus production. Therefore, although MORC3 is required for effective repression of the MIEP to establish latency, cells that express IE proteins do not proceed through a full lytic cycle.

### 3.5 MORC3 forms PML‐independent NBs in mononuclear cells

**Figure 6:**
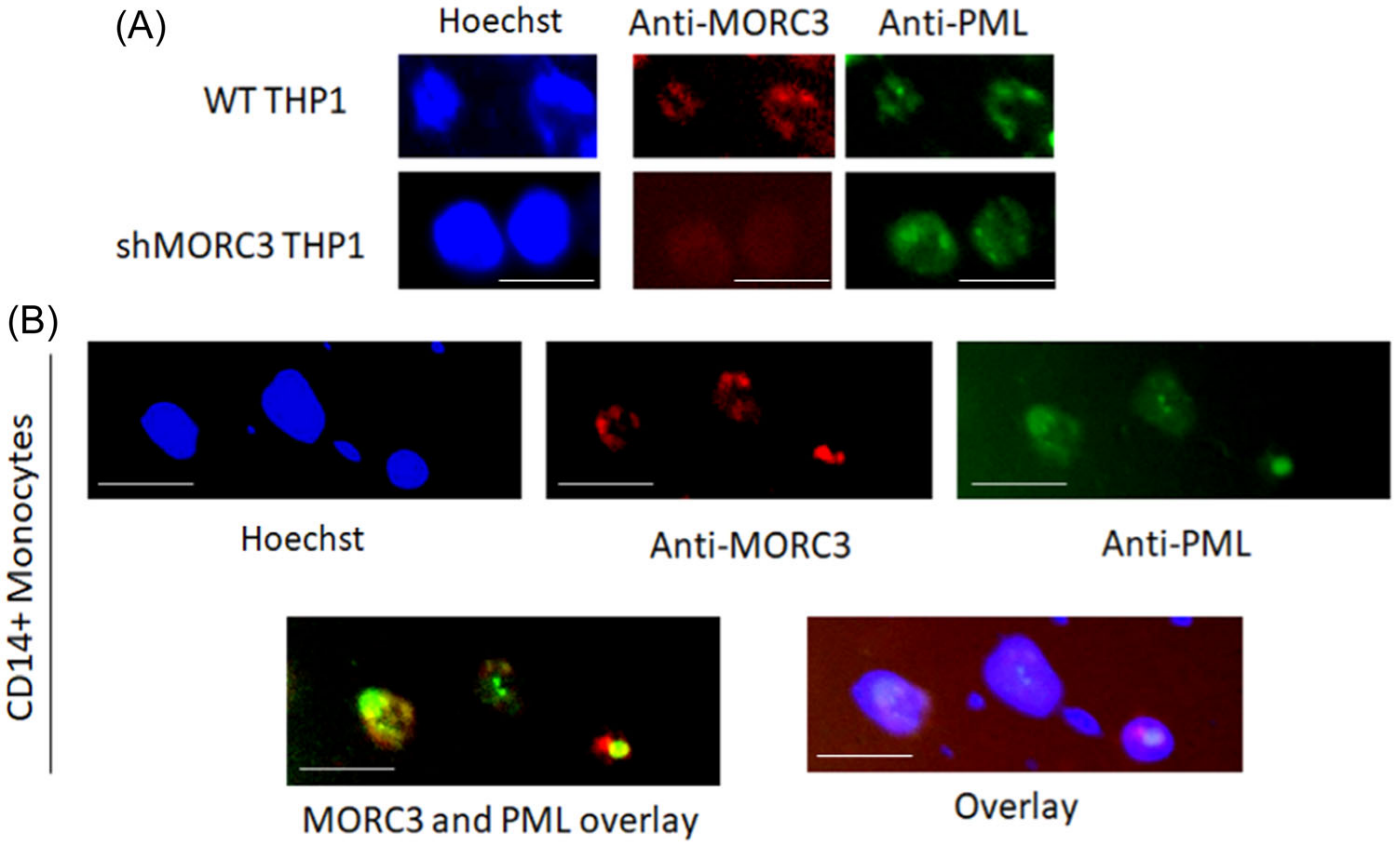
MORC3 forms PML‐independent bodies in THP1 and primary monocyte cells. WT THP1 cells, shMORC3‐THP1 cells, or primary CD14+ monocytes were stained with Hoechst (blue), anti‐MORC3‐594 (red) and anti‐PML‐488 (green) where the white line represents 20 µM (A and B). Overlays of PML and MORC3 and PML, MORC3 and Hoechst are also shown (B).

Higher magnification of the MORC3 fluorescence images in Figure 5A showed that MORC3 appeared to form NBs in WT THP1 cells. As it has previously been shown that MORC3 can be a component of PML NBs, we tested whether the MORC3 NBs identified in the THP1 cells co‐localized with PML NBs. Figure 6A shows that some MORC3 NBs do co‐localize with PML bodies in WT THP1 cells but that there are also MORC3 NBs which are not associated with PML. As expected, shMORC3 cells did not contain visible MORC3 NBs but did contain PML NBs (Figure 6A) suggesting that PML NB formation in THP1 cells is not dependent upon the presence of MORC3. Analysis of primary CD14+ monocytes also showed that both PML and MORC3 NBs could be detected and that some co‐localized but others did not (Figure 6B). This is consistent with previous studies in HeLa and murine embryonic fibroblast (MEF) cells which have shown that MORC3 can form PML‐independent NBs.^15^ We now show that myelomonocytic cells also form MORC3 NBs that are distinct from PML NBs.

### 3.6 HCMV latency causes PML NB disruption but enhances MORC3 NBs

**Figure 7:**
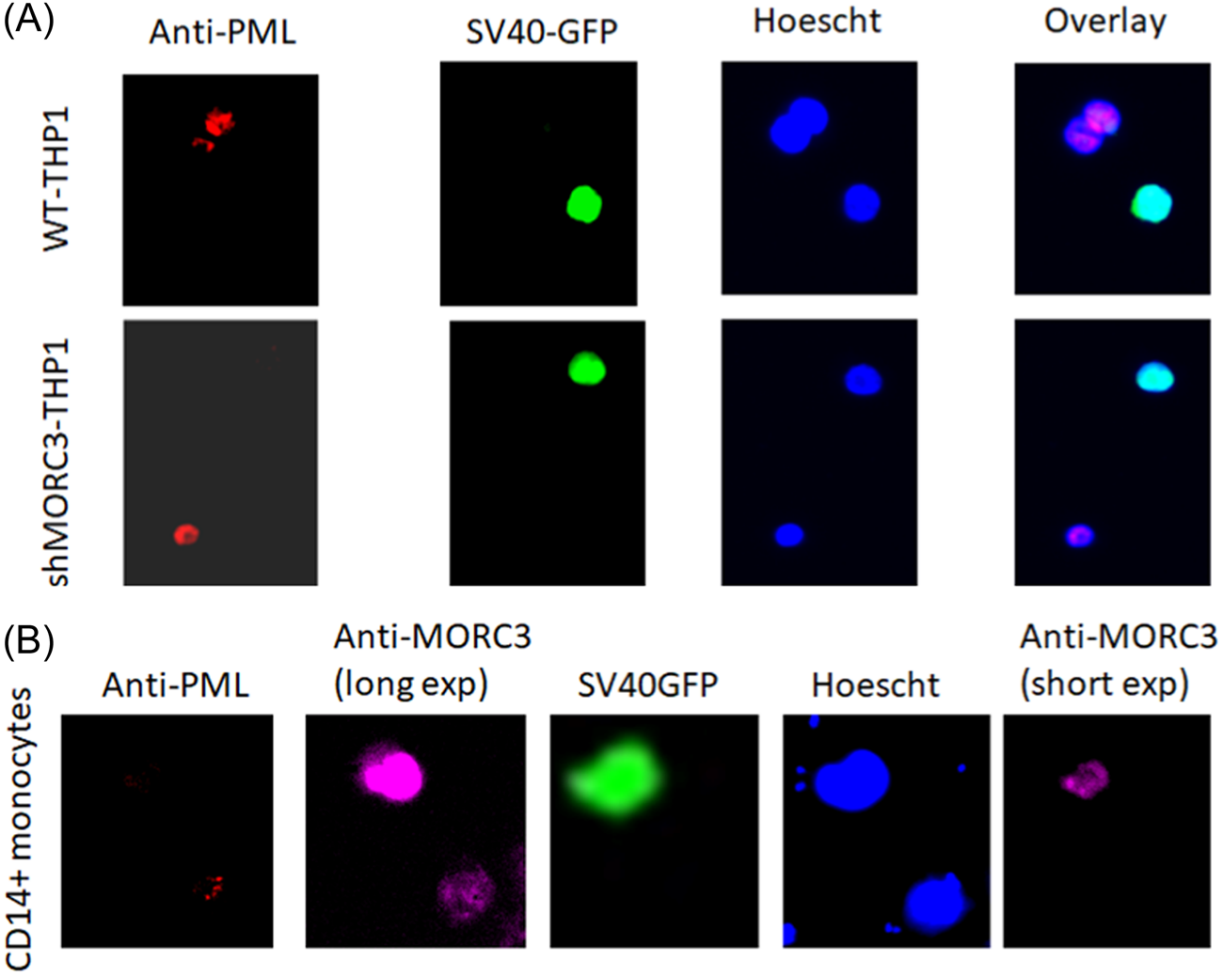
PML NBs are disrupted during latency in the presence of absence of MORC3 but MORC3 is upregulated during latency, forming MORC3 NBs. WT, shMORC3‐THP1 (A) or CD14+ primary monocytes (B) cells were latently infected with TB40E‐SV40GFP virus (green) for 4 days before staining with anti‐PML antibody (red, A and B), anti‐MORC3 antibody (magenta, B), Hoechst 33324 (blue, A and B) and visualized for GFP (green, A and B), where the white line represents 20uM, by fluorescence microscopy.

We have previously shown that PML bodies are disrupted by latency‐associated expression of viral LUNA during HCMV latent infection.^37^ Therefore, we initially tested whether latency‐associated targeting of MORC3 was needed for PML disruption in latently infected cells. Figure 7A shows that in the presence or absence of MORC3, PML bodies are disrupted in latently infected THP1 cells (those expressing GFP after infection with TB40E‐SV40GFP virus). Therefore, the disruption of PML bodies during latency is independent of MORC3.

We have observed that MORC3 is upregulated during HCMV latency (Figures 2C and 4A–D) and that MORC3 is required for optimal repression of the MIEP (Figure 5B,C). Therefore, we next tested whether MORC3 NBs were disrupted during HCMV latency to redistribute MORC3 to alternative sites, perhaps for repression of the MIEP, or whether the MORC3 NBs remained intact in the presence of HCMV latent infection. Figure 7B confirms that, in latently infected CD14+ primary monocytes, there is an upregulation of MORC3 but, additionally, PML NB disruption and that this is in the absence of MORC3 disruption. This is further clarified, using a lighter exposure of this figure (Figure 7B, short exp), where it is evident that, whilst PML NBs are disrupted, the MORC3 NBs are still present in the latently infected cells. Taken together, these data confirm that, whilst during latency PML NBs are disrupted, MORC3 is upregulated and MORC3 NBs are still present in latently infected cells begging the question as to whether MORC3 NBs are directly involved in control of MIEP activity during latency.

### 3.7 MORC3 associates with the MIEP

**Figure 8:**
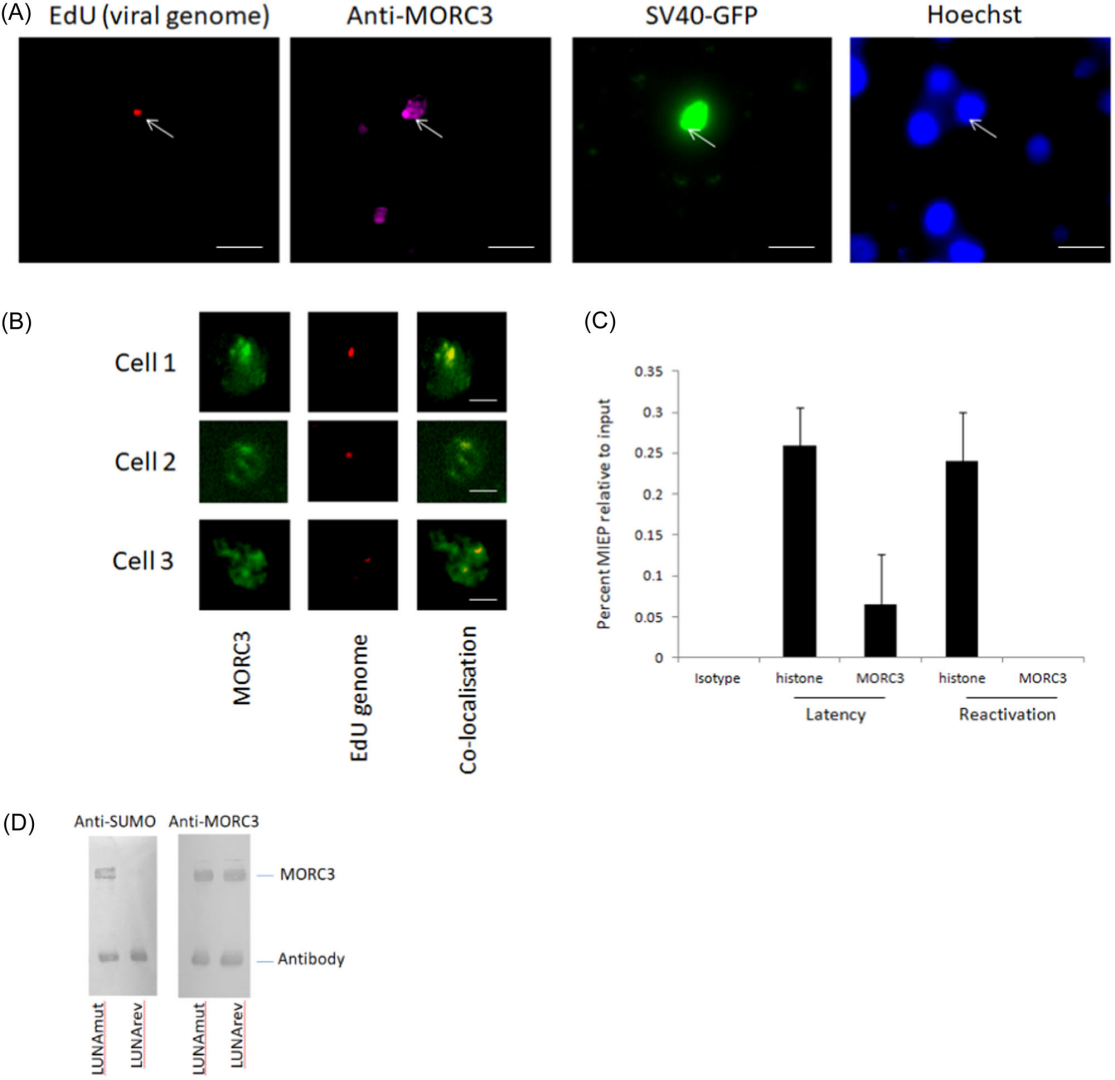
HCMV genome co‐localizes with MORC3 during HCMV latency and MORC3 associated with the MIEP. Primary CD14+ monocytes were infected with EdU‐labeled TB40E‐SV40GFP virus (green) for 4 days before staining with anti‐MORC3 (magenta), EdU (red), and Hoechst 33324 (blue) where the white line represents 20 µM (A). Alternatively, cells were stained for MORC3 (green) and EdU (red) and analyzed for co‐localization where the white line represents 10 µM (B). Finally, CD14+ monocytes that had been infected with TB40E‐SV40GFP for 4 days were harvested for ChIP analysis with anti‐MORC3 or anti‐histone and isotype control antibodies. Data shown represents triplicates with standard deviation about the mean (C). Finally, CD14+ monocytes were infected with a sumoylase deficient LUNA (LUNAmut) or the revertant virus (LUNArev) and the immunoprecipitated with anti‐MORC3 antibody. Samples were harvested for western blot analysis with either SUMO (anti‐SUMO) or MORC3 (anti‐MORC3) (D).

Given that MORC3 is upregulated during latency, that MORC3 NBs are present during latent infection and that MORC3 is known to repress the MIEP, we next tested whether MORC3 or MORC3 NBs interact with viral genomes in latently infected cells. To do this we, first, visualized the location of viral genomes during latent infection with respect to MORC3 NBs. Figure 8A shows that EdU‐labeled viral genomes (red) are detectable in cells latently infected with EdU‐labeled TB40E‐SV40GFP virus (green) and that these viral genomes appeared to co‐localize with MORC3 NBs (magenta).

To analyze this in more detail, and confirm this colocalization of viral genome with MORC3 NBs, we re‐analyzed the localization of EdU‐labeled viral genome (again in red) with MORC3 (now shown in green) to assist detection of any colocalization (yellow). Figure 8B shows some examples where the viral genomes in latently infected cells do co‐localize with MORC3 NBs (we observed similar effects in multiple independent fields of view). Next, to address whether MORC3 associates with the MIEP itself, ChIP assays were carried out. Figure 8C shows that MORC3 is, indeed, associated with the MIEP in latently infected monocytes but, perhaps not unexpectedly, this association is lost upon virus reactivation when the latency‐associated repressive effects of MORC3 would need to be reversed. These data suggest that the MORC3 upregulated during associates with the MIEP, however, MORC3 has many functions which are regulated by post translational modification. Pertinent to our study deSUMOylated MORC3 can form PML‐independent NBs and act as a molecular clamp, able to bind to DNA.^20^ Therefore, we next asked whether during HCMV latency there is a mechanism in place to cause MORC3 to be deSUMOlyated to drive association with DNA. The latency associated viral protein, LUNA, is known to have deSUMOylase function so we next tested whether LUNA could cause the deSUMOylation of MORC3. Figure 8D shows, by immunoprecipitation, that in the absence of deSUMOylase‐competent LUNA, HCMV does not cause deSUMOylation of MORC3 but when the LUNA deSUMOylase is functional, MORC3 is not SUMOylated during latency in primary CD14+ cells.

These data are consistent with previous analyzes which have suggested that deSUMOylated MORC3 tends to form PML independent NBs, and, in its deSUMOylated form where it is proposed to act as a molecular clamp, it functions to mediate MIEP repression. In essence, MORC3, likely as MORC3 NBs, represses viral IE gene expression during latency by targeting and binding to the viral MIEP.

## 4 CONCLUSIONS

The data presented here demonstrate that MORC3 plays an important role in repressing viral MIEP activity to establish and maintain HCMV latency. This is in contrast to lytic infection where MORC3 is degraded to enhance MIEP activity.^21^, ^24^ Thus we propose a balanced model of MORC3 upregulation during latency and downregulation during reactivation/lytic infection. This model is complicated by a second function of MORC3 whereby loss of MORC3 results in the activation of innate immunity. This was demonstrated with HSV‐1, whereby ICP0 induced degradation of MORC3 triggered interferon beta (IFN‐beta) expression. This was explained by the capacity of MORC3 to bind to the IFN‐beta promoter to silence gene expression, which was lost upon degradation. Essentially, this was proposed as a fail‐safe against manipulation by pathogen infection.^38^ Whether the downregulation of MORC3 in the context of cellular differentiation is less clear—it is not reported that differentiating myeloid cells spontaneously start expressing IFN‐beta so this may be a moot point in the context of viral reactivation. Furthermore, we and others have shown that latent viral gene products regulate components of the innate immune system^37^, ^39^, ^40^, ^41^, ^42^ which may also serve to counteract this additional function of MORC3.

The focus of this study was to understand the role of MORC3 upregulation and activity in the establishment and maintenance of latency. SUMOylated MORC3, associated with PML, is known to mediate transcriptional repression in a Daxx‐dependent manner.^19^, ^20^, ^21^ However, our data suggest that MORC3‐mediated repression of the MIEP during latency is unlikely to involve SUMOylated MORC3 or its interaction with PML/Daxx for a number of reasons. For instance, one important viral gene product expressed during latency, LUNA, is a known viral de‐SUMOylase and, consistent with this, latent infection results in de‐SUMOylation of MORC3; this MORC3 de‐SUMOylation is not observed in latent infection with viruses lacking LUNA. Additionally, PML bodies are disrupted during latency by LUNA and some reports have shown that Daxx is not required for latency‐associated repression of the MIEP.^43^, ^44^ Instead, our data suggests that the upregulation of MORC3 during latency and its de‐SUMOylation by LUNA drives MORC3 NB formation. These MORC3 NBs, which form independently of PML NBs and which can form molecular clamps to the DNA,^20^ facilitate the silencing of viral genomes during latency.

Thus, through the activity of LUNA HCMV has simultaneously disabled a host mechanism potentially inhibitory to viral reactivation (PML bodies) whilst enhancing a repressive function that supports viral latency through inhibition of the MIEP. However, it is noteworthy that loss of MORC3 from cells that are normally non‐permissive did not result in a full lytic infection but, instead, led to an abortive infection. These data suggest that MORC3 is important for promoting latency via silencing of the MIEP but could also be important for ensuring that IE gene expression does not occur in undifferentiated myeloid cells that cannot support viral replication and thus evade IE detection by immune cells.^45^ The in vivo importance of this could be to prevent immune recognition by the prodigious T cell response directed against HCMV much the same as has been proposed for the role of microRNAs.^46^ Thus repression of the MIEP not only regulates timely viral gene expression but aids immune evasion of the virus during latency.

Many viruses target PML bodies for disruption via different mechanisms during their infection cycles. For instance, during lytic infection, HSV‐1, Kaposi's Sarcoma Herpes Virus (KSHV) and Epstein Barr Virus directly degrade PML via viral proteins such as ICP0, ORF75 and BNRF1,^47^, ^48^, ^49^ respectively, though there is no evidence that PML NBs are disrupted by these viruses during latent infection. In contrast, HCMV disrupts PML NBs during both lytic and latent infection but via different mechanisms. During lytic infection, the viral IE72 protein disrupts PML NBs ^50^ to activate the MIEP and facilitate transcription of viral genes during lytic infection. However, during latent infection, in the absence of IE72 expression, PML bodies are also disrupted but this is mediated by the de‐SUMOylase activity of LUNA.^37^ Current thinking would suggest that, during herpesvirus latency, factors that repress lytic gene expression would be retained rather than degraded, as is the case for HSV‐1, EBV and KSHV. However, during HCMV latency, PML NBs are disrupted by LUNA‐mediated PML de‐SUMOylation and it has been argued that this is to ensure speedy reactivation.^37^ Thus the mechanisms which regulate PML NBs are likely working in balance with MORC3 NBs which are not disrupted as readily.

Perhaps counterintuitively this absence of PML bodies during latency is not, in itself, sufficient for reactivation of the HCMV MIEP; infection of undifferentiated myeloid cell lines in which PML has been knocked‐out still results in MIEP repression and the establishment of latency. We show, here, that this may be due, at least in part, to the ability of latent infection to upregulate MORC3 which, in turn, forms MORC3 NBs. We believe that this is likely accompanied by deSUMOylation of MORC3 by LUNA which may also enhance the ability of MORC3 to form a molecular clamp and associate with the MIEP on the viral genome. These repressive MORC3 NBs function to aid repression of the MIEP during latency in the absence of PML NBs. How the MORC3 NBs are removed before reactivation is not yet known but it is known that during lytic infection, MORC3 is degraded, although again the viral protein required for this is not known.^21^ Interestingly, MORC3 is also degraded during HSV‐1 infection via the viral protein ICP0^22^ thus degradation of MORC3 during lytic infection may be a common function to herpesviruses. However, this is the first report of any herpesviruses upregulating MORC3 to aid repression during latency.

## AUTHOR CONTRIBUTIONS

**Anna Champion**: performed experiments. **Alexandra Rowland**: generated the epigenetic library. **Levia Yee**: performed experiments, DB performed and designed experiments. **Matthew Reeves**: wrote the manuscript. **Paul Lehner**: designed experiments and edited the manuscript. **John Sinclair**: edited the manuscript and designed experiments. **Emma Poole**: performed and designed experiments and wrote the manuscript.

## CONFLICT OF INTEREST STATEMENT

The authors declare no conflicts of interest.

## ETHICS STATEMENT

All research describing studies on primary human material with HCMV were assessed and approved by the Cambridge Local Research Ethics committee. Informed consent was received from blood donors with the Cambridge Local Research Ethics committee and the Cambridge Internal Review Board. Cells were harvested from healthy adult donors, and the decision to use tissue was not affected by gender and age, as this was not important to the studies performed. The patients/participants provided their written informed consent to participate in this study.

## Supporting information

Supporting information.

Supporting information.

## Data Availability

The data that supports the findings of this study are available in the Supporting Information material of this article. The original contributions presented in the study are included in the article/Supporting Information material, further inquiries can be directed to the corresponding author. All associated data is available within supplementary data in the manuscript.
