# Supplementary material for: MORC3 represses the HCMV major immediate early promoter in myeloid cells in the absence of PML nuclear bodies
Source: J Med Virol. 2023 Nov 27;95(11):e29227. doi: 10.1002/jmv.29227 (PMC10952291; doi:10.1002/jmv.29227)
Supplement: Supplementary file 1 — Supporting information. [file JMV-95-0-s001.docx]

**Supplemental materials**

**Plasmids for CRISPR screen**

| **Plasmid** | **Description** | **Origin** |
| --- | --- | --- |
| pHRSIN.pSFFV FLAG-NLS-CAS9-NLS pSV40 Blast | pSp. CAS9, FLAG-tagged | ^51^ |
| pKLV.U6 pGK Puro-2A-BFP | sgRNA vector, a kind gift from Kosuke Yusa (Wellcome Trust Sanger Institute, UK) | ^52^ |

**sgRNA for CRISPR screen**

| **sgRNA** | **Sequence** | **Vector** | **Ref.** |
| --- | --- | --- | --- |
| β2m sg1 | 5’-GGCCGAGATGTCTCGCTCCG-3’ | pKLV.U6 pGK Puro-2A-BFP | ^53^ |

**Primers for CRISPR screen**

| **Primer** | **Sequence** | **Usage** |
| --- | --- | --- |
| Libr PCR1 FW | 5’-GCTTACCGTAACTTGAAAGTATTTCG-3’ | PCR1 for integrated sgRNA in CRISPR screen |
| Libr PCR1 RV | 5’-CGAGACTAGTGAAACGTGCTAC-3’ | PCR1 for integrated sgRNA in CRISPR screen |
| Libr PCR2 FW | 5’-AATGATACGGCGACCACCGA GATCTACACTCTCTTGTGGAAAGGACGAAACACCG-3’ | PCR2 for integrated sgRNA in CRISPR screen |
| Libr PCR2 RV | 5’-CAAGCAGAAGACGGCATACGAGATnnnnnnnGTGACTGGAGTTCAGACGTGTGCTCTTCC  GATCT CCATTTGTCACGTCCTGCACG-3’ | PCR2 for integrated sgRNA in CRISPR screen,  nnnnnn indicates variable Illumina index sequence |
| Libr seq | 5’- CTTGTGGAAAGGACGAAACACCG-3’ | Illumina sequencing of CRISPR screen |
